# 3D-Printing-Assisted Fabrication and Characterization of Pregabalin-Loaded PVA/PVP Dissolving Microneedle Arrays

**DOI:** 10.3390/mi17060676

**Published:** 2026-05-29

**Authors:** Arjun Gokulan Manivannan, Sreeja Balakrishna Pillai Suseela, Mohana Priya Kandan, Narayanan Jayshankar, Bhupendra G. Prajapati, Chitra Vellapandian, Suhaskumar Patel, Dignesh Khunt

**Affiliations:** 1 Department of Pharmacology, SRM College of Pharmacy, Faculty of Medicine and Health Sciences, SRM Institute of Science and Technology, Kattankulathur, Chengalpattu 603203, Tamil Nadu, India; 2 Department of Biomedical Engineering, CEG Campus Anna University, Chennai 600025, Tamil Nadu, India; 3 Parul Institute of Pharmacy, Faculty of Pharmacy, Parul University, Waghodia, Vadodara 391760, Gujarat, India; 4 Centre for Research Impact & Outcome, Chitkara College of Pharmacy, Chitkara University, Rajpura 140401, Punjab, India; 5 School of Pharmacy, Gujarat Technological University, Gandhinagar 382028, Gujarat, India

**Keywords:** microneedles, dissolving microneedles, pregabalin, 3D printing, PVA/PVP, transdermal drug delivery, PDMS, drug delivery systems

## Abstract

**Background:** A transdermal drug delivery system has significant benefits over conventional routes; however, its effectiveness is limited by the barrier properties of the stratum corneum. Dissolving microneedles (DMNs) have emerged as a minimally invasive strategy to enhance drug permeation while improving patient compliance. The integration of advanced fabrication techniques such as 3D printing enables precise control over microneedle geometry and reproducibility. **Objective:** This study aimed to fabricate and characterize pregabalin-loaded PVA/PVP dissolving microneedle arrays using a 3D-printing-assisted mold fabrication approach for efficient transdermal drug delivery. **Methods:** Microneedle master molds were fabricated using 3D printing, followed by replication using polydimethylsiloxane (PDMS) to obtain negative molds. Pregabalin-loaded bilayer microneedles were prepared using a micromolding technique with PVA/PVP polymers. The formulation was evaluated through rheological analysis, scanning electron microscopy (SEM), mechanical strength testing, insertion studies, swelling behavior, drug loading efficiency, Fourier transform infrared spectroscopy (FTIR), differential scanning calorimetry (DSC), X-ray diffraction (XRD), and in vitro drug release studies. **Results:** The fabricated microneedles exhibited uniform geometry with sharp tips and no structural defects. Rheological analysis confirmed shear-thinning behavior suitable for mold filling. The microneedles demonstrated adequate mechanical strength (~3.3 N/needle) and efficient insertion into the parafilm model. Drug loading efficiency was high (92.4%), indicating effective encapsulation. FTIR analysis confirmed compatibility between drug and polymers, while DSC and XRD results indicated partial amorphization of pregabalin within the polymer matrix. The formulation showed a biphasic drug release profile with an initial burst followed by sustained release, achieving ~96.8% cumulative release over 24 h. **Conclusions:** The study successfully demonstrates a robust and reproducible 3D-printing-assisted approach for fabricating pregabalin-loaded dissolving microneedles. The developed system exhibited desirable mechanical, physicochemical, and drug release properties, highlighting its potential as an effective transdermal drug delivery platform.

## 1. Introduction

The transdermal drug delivery system has evolved as a new approach for the management of topical and systemic diseases. Transdermal delivery provides diverse strategies compared to oral and parenteral routes, offering various benefits such as circumventing first-pass metabolism, gastrointestinal degradation, and injection-related discomfort and allowing controlled and sustained release of drugs [1]. Although it has various benefits, it lacks permeation of the stratum corneum, the major layer of dense lipid protein acting as a barrier to the skin, which restrains the entry of most hydrophilic substances. Thus, it requires penetration enhancers that mediate the permeation of the substance into the skin without causing disturbance and damage to the barrier layer [2]. Conventional transdermal delivery systems such as patches, ointments, and hydrogels primarily rely on passive diffusion across the stratum corneum. Although these systems offer advantages such as ease of application and sustained drug release, their effectiveness is significantly limited by the barrier properties of the stratum corneum, particularly for hydrophilic and high-molecular-weight drugs. In contrast, microneedle-based systems utilize a physical permeation mechanism by creating transient microchannels in the skin, thereby bypassing the stratum corneum and enabling enhanced drug delivery. This fundamental difference in permeation mechanism allows microneedles to achieve improved drug bioavailability and delivery efficiency compared to conventional transdermal approaches. Despite the availability of several conventional transdermal enhancement approaches, including chemical enhancers, iontophoresis, and ultrasound-assisted delivery systems, achieving efficient and controlled permeation of therapeutic agents across the stratum corneum remains a significant challenge [3]. In contrast, microneedles (MNs) have emerged as a promising minimally invasive transdermal delivery platform capable of creating transient microchannels in the skin, thereby improving drug permeation without causing significant pain or tissue damage. Microneedles are widely investigated for the delivery of various therapeutic agents owing to their ability to enhance patient compliance, bypass first-pass metabolism, and facilitate controlled drug delivery [4]. Pregabalin is widely prescribed for the management of neuropathic pain, epilepsy, and generalized anxiety disorders. Despite its therapeutic efficacy, pregabalin is primarily administered orally and requires frequent dosing due to its relatively short biological half-life, which may lead to fluctuations in plasma drug concentration and dose-related adverse effects such as dizziness, somnolence, and gastrointestinal discomfort. In addition, repeated oral administration may reduce patient compliance during long-term therapy. These pharmacokinetic limitations make pregabalin a suitable candidate for sustained transdermal drug delivery approaches, including dissolving microneedle systems. Traditional microneedle fabrication techniques, including photolithography, laser cutting, micromolding, and etching methods, have been extensively explored for the development of transdermal delivery systems. However, these approaches often require sophisticated instrumentation, multistep fabrication procedures, high production costs, and limited flexibility in achieving customized microneedle geometries, which may affect fabrication reproducibility and scalability.

Various types of MN are being developed, such as solid, hollow, coated, hydrogel-type, and dissolving MNs. Among these, dissolving MNs are highly integrated due to their benefits of increased biocompatibility, reliability, and evasion of biohazardous waste. Swellable and water-soluble polymers are used in drug encapsulation, and upon insertion these needles readily dissolve, releasing the drug into the skin. Hence, the choice and utilization of polymers play a major role in their fabrication. Recent studies have discovered the promising polymer must adhere to these characteristics to integrate hydrophilic polymers, hydrogel matrices, and functional nanocarriers to enhance the mechanical strength and dissolution properties [5,6].

Configuration of these MNs highly impacts the consistency, reliability, geometry, texture, and performance of the MNs. The traditional developmental process of MNs is a stringent technique but lacks scalability and is substantially cost-intensive. The micromolding-assisted polymer casting technique has been highly developed in recent years and offers simplicity and adaptability. The amalgamation of nanoparticles and natural polymers widely enhanced the application of MNs in varied fields [4,6,7]. The embedding of polymers such as PVA and PVP increases the rigidity, elasticity, and dissolution rate of the fabricated MN [8]. Recent advancements in 3D printing technologies have provided new opportunities for microneedle fabrication by enabling rapid prototyping, precise geometrical control, improved reproducibility, and customizable design configurations. Several studies have demonstrated the potential of 3D-printing-assisted fabrication approaches for developing structurally optimized dissolving microneedle systems with enhanced mechanical and drug delivery properties.

3D printing has modernized the generation of MN molds, which enables personalizable crafting with regulation of microneedle height, interspacing, and tip geometry. Studies have recently marked the development of 3D-printed master molds for delivering drugs such as diclofenac sodium through hydrogel-based MNs, which showcased efficient reproducibility and precision [1]. Further studies are characterized by the structuring of 3D-engineered templates, which focuses on unifying 3D-printed molds in the development of dissolving microneedle systems. Although dissolving microneedle systems for pregabalin delivery have been previously explored, limited studies have investigated the integration of 3D-printing-assisted mold fabrication with bilayer PVA/PVP polymeric systems to improve fabrication reproducibility, mold precision, and controlled drug release characteristics. In the present study, a 3D-printing-assisted fabrication strategy was utilized to develop structurally optimized pregabalin-loaded dissolving microneedles with enhanced geometrical consistency and reproducible fabrication. In addition, the bilayer polymeric design was intended to facilitate efficient drug loading in the needle tips while maintaining mechanical stability in the baseplate, thereby contributing to controlled biphasic drug release behavior [7,8,9,10].

The anticipated progress of MNs could fulfill the rising need for improved alternatives for transdermal delivery systems. Despite significant advancements in dissolving microneedle systems, several limitations remain, including poor reproducibility of microneedle geometry, limited control over fabrication precision, and challenges in achieving controlled and sustained drug release. Conventional micromolding techniques often suffer from variability in needle structure and drug distribution, which can affect performance and reliability.

In this context, the present study introduces a 3D-printing-assisted mold fabrication approach combined with a bilayer PVA/PVP dissolving microneedle system for pregabalin delivery. The use of 3D printing enables precise control over microneedle geometry, including height, tip sharpness, and interspacing, thereby improving reproducibility and structural consistency. Additionally, the bilayer design facilitates efficient drug loading in the needle tips while maintaining mechanical strength in the baseplate, contributing to a controlled biphasic drug release profile.

To the best of our knowledge, this study represents a novel integration of 3D-printing-assisted mold fabrication with a bilayer polymeric microneedle system specifically optimized for pregabalin delivery, addressing key limitations associated with conventional fabrication methods and enhancing the overall performance of dissolving-microneedle-based transdermal drug delivery systems.

## 2. Material and Methods

### 2.1. Materials

Pregabalin (≥99.0%), polyvinylpyrrolidone (PVP), polyvinyl alcohol (PVA), glycerol (99.5%), Parafilm^®^ M (~ 130 µm thickness) and phosphate-buffered saline (PBS) (pH 7.4) were purchased from Sigma Aldrich (India). All the other reagents used were of analytical grade.

### 2.2. Methods

#### 2.2.1. Design and Fabrication of 3D-Printed Master Mold and Reverse PDMS Microneedle Mold

**Figure 1:**
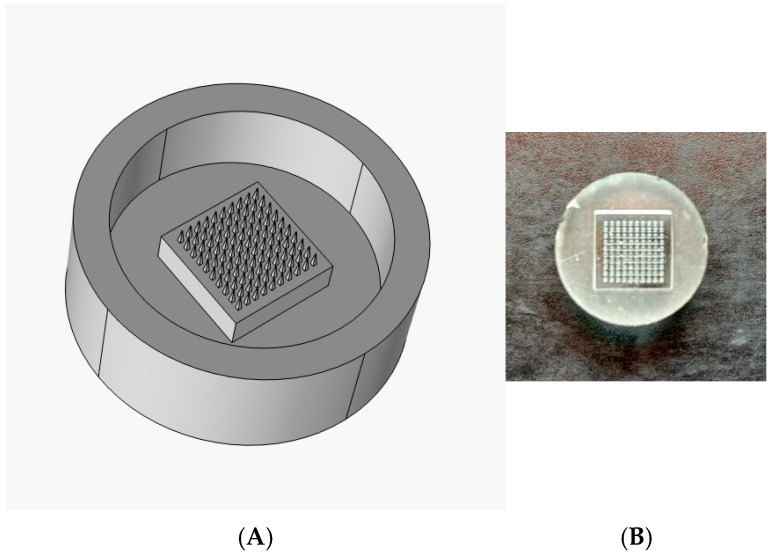
(**A**) 3D-designed master microneedle mold showing geometrical specifications. (**B**) Fabricated reverse PDMS microneedle mold obtained from the 3D-printed master mold.

**Figure 2:**
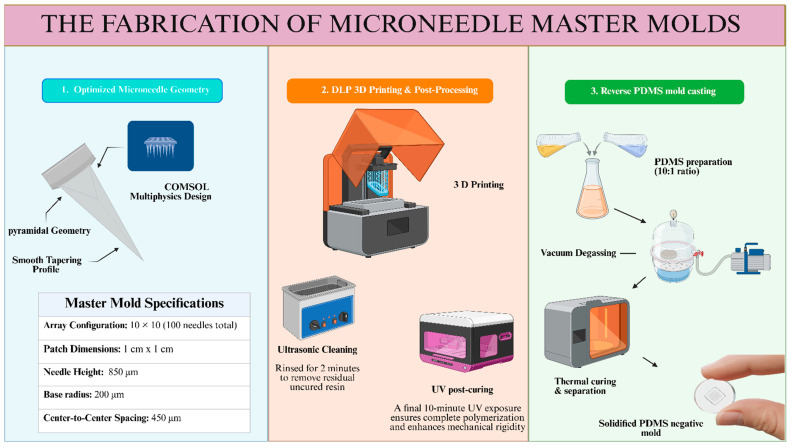
Schematic illustration of the fabrication process of pregabalin-loaded bilayer dissolving microneedle patches using a 3D-printing-assisted mold fabrication approach.

An SLA 3D printer (Formlabs) was used for the fabrication of the master micromold. Micromold masters were designed using COMSOL Multiphysics software version 6.3 with the following geometries: conical-shaped with a height of 850 µm, a bottom radius of 200 µm, and a top radius of 15 µm. The designed microneedle patch comprised 10 × 10 needles with 450 µm interspacing between the needles, as shown in Figure 1A. After designing microneedle patches, the CAD files were converted into STL files. The STL files were then loaded into Preform Software version 3.56, which is associated with the Formlabs 3D printer, to prepare the micromold masters. After 3D printing, the master molds were washed in an ultrasonic washer in a dedicated cleaner (Formwash) with isopropyl alcohol for 10 min until the excess resin was removed. Following this, the molds were cured with a UV lamp (Form Cure) [1,11]. Figure 2 shows the schematic representation of the process.

The polydimethylsiloxane (PDMS) MN molds for dissolving microneedle patches were fabricated by using 3D-printed master molds using the liquid phase of PDMS mixed with a curing agent in a ratio of 10:1. Following this, the PDMS mixture was poured into the 3D-printed master mold, and a vacuum pump was utilized to remove the air bubbles, and then it was kept in a hot air oven to cure the PDMS mold at 60 °C for 4 h. Finally, an inverse PDMS mold was carefully removed from the 3D-printed master mold, as shown in Figure 1B [1,4,8].

#### 2.2.2. Fabrication of Pregabalin-Loaded Bilayer Dissolving Microneedle Patches

**Figure 3:**
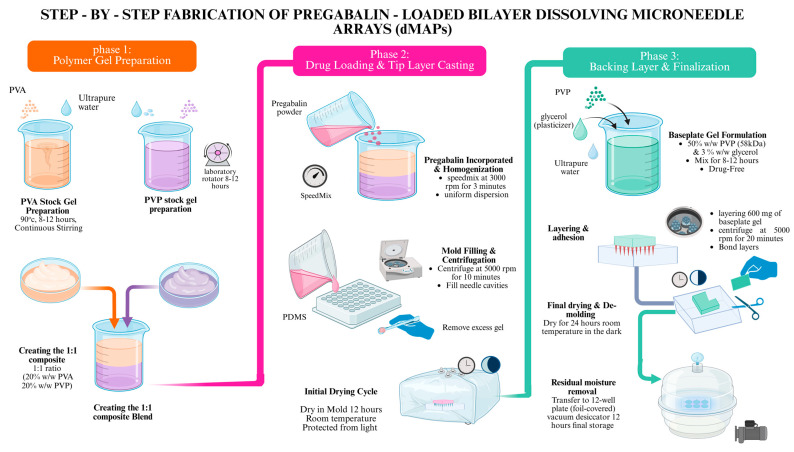
Schematic illustration of the fabrication process of pregabalin-loaded bilayer dissolving microneedle patches.

The pregabalin-loaded bilayer dissolving microneedles were fabricated using the micromolding method. In this study, the dissolving microneedles were fabricated as a bilayer system consisting of a drug-loaded tip layer (PVA 20% *w*/*w* + PVP 20% *w*/*w* + pregabalin) and a drug-free baseplate (PVP 50% *w*/*w* + glycerol 3% *w*/*w*). For the preparation of MN tips, a tip-casting gel was formulated by thoroughly mixing 5 mg of pregabalin, 100 mg of PVA-PVP gel, and 100 mg of water. A small quantity of the tip-casting gel was poured into the PDMS reverse mold and centrifuged at 5000 rpm for 15 min to fill the cavities and remove the air bubbles, and the excess gel was removed. Then the mold was left to dry for 12 h in the dark at room temperature. Subsequently, baseplate polymeric gel was carefully poured over the tip layer, centrifuged at 5000 rpm for 20 min, and left to dry in the dark at room temperature. After 24 h, the pregabalin-loaded bilayer dissolving microneedles were carefully removed from the mold; side walls were removed carefully using scissors. Finally, the dissolving MNs were further dried in a vacuum desiccator for 12 h to remove any residual moisture. The theoretical drug loading per patch was approximately 5 mg, and uniform drug distribution was ensured through thorough mixing and centrifugation-assisted mold filling [2,12]. Figure 3 shows the schematic representation of the process.

### 2.3. Characterization of Dissolving Microneedle

#### 2.3.1. Rheology Analysis (Viscosity)

The rheological behavior of PVA solution, PVP solution, and pregabalin-loaded PVA/PVP polymeric solution was assessed using a Brookfield rheometer (R/S-CPS, P25 DIN plate, Brookfield Engineering Laboratories, Middleboro, MA, USA) at 25 ± 1 °C. Measurements were carried out over a shear rate range of 0.1–100 s^−1^ at a plate diameter of 25 mm and a gap of 1 mm. Upon each measurement, 0.6 mL of sample was utilized to examine the rheological parameter. Polyvinyl alcohol (PVA, molecular weight ~85,000–124,000) and polyvinylpyrrolidone (PVP, K30 grade) polymer solutions, as well as pregabalin-loaded polymeric blends, were analyzed under identical conditions [13].

#### 2.3.2. Morphology (SEM)

To assess the morphological characteristics of MNs, like needle height, base diameter, tip sharpness, and aspect ratio, field emission scanning electron microscopy (FESEM) (JEOL, IT800HL) was performed at an accelerating voltage of 15 kV under low-vacuum conditions. The MN patches were sectioned and mounted onto an aluminum stub with double-sided adhesive conductive carbon tape to ensure firm placement. Then the MNs were carefully coated using gold sputter for about a minute to enhance the surface conductivity and were further imaged. The FESEM micrographs were visualized at 30x magnification [6,13,14].

#### 2.3.3. Uniformity of Thickness and Width

The thickness of the patches was analyzed using a vernier caliper to ensure its uniformity. Measurements were performed using three independent microneedle patches (n = 3), and values were reported as mean ± standard deviation. The weight variation among the patches was examined by weighing the patches individually using an electronic digital balance; the mean value ± standard deviation was derived. Both tests were performed in triplicate [15].

#### 2.3.4. Folding Endurance

The folding endurance was determined by holding the patch with forceps and folding until the patch ruptured. The number of folds required to cause rupture is recorded, which indicates the folding endurance of the object. The mean value and standard deviation are derived [15,16]. This test was performed to evaluate the flexibility and mechanical durability of the microneedle patch during handling and application.

#### 2.3.5. Swelling Study

The swelling study was conducted to evaluate the hydration behavior of the polymeric microneedle matrix in phosphate-buffered saline (PBS, pH 5.5). The MN patches are completely dried in an oven until reaching 3 equal consecutive weights, taken as their initial weight (Wo). The microneedle patch was immersed in phosphate-buffered saline (PBS, pH 5.5) and incubated at 37 °C. At equal intervals the patch is removed and blotted using tissue on the surface of the patch to remove excess water, and the patch is weighed gently (Wt). The swelling index is calculated using the formula SI = (W_t_ − W_0_)/W_0_ × 100. This calculation reflects the swelling ability of the patch and can also estimate the period taken to reach equilibrium swelling [5]. This study provides insight into the hydration behavior of the polymer matrix, which influences microneedle dissolution and drug release characteristics.

#### 2.3.6. Mechanical Strength (Axial Compression)

The axial compression test is to analyze the force required for an individual needle to deform or fracture using an applied axial load (digital force gauge (ZTA-50N)). The MN patch is secured onto the platform using a double-sided tape for firm positioning. A flat stainless-steel probe (2–10 mm diameter) is vertically lowered onto the patch with a low trigger force (~0.05 N). The probe is lowered at a controlled speed (typically 0.5–1 mm/s) onto the MN. The instrument records the force displacement onto the MN; a sudden drop in the force onto the MN indicates the rupturing of the MN, and this value sets the maximum force limit or fracturing force. To obtain the force per needle, the maximum force exerted is divided by the number of needles ruptured. The heights of the needle prior to and after the compression are compared using optical microscopy to estimate the percentage of deformation of the needle height. The test is performed to determine the fracture force, needle structural integrity under axial load, deformation behavior, and suitability of MNs for insertion through the stratum corneum [17].

#### 2.3.7. Tensile Strength

The TAXT Plus Texture Analyzer fitted with A/TG tensile grips is equipped to analyze the tensile strength of the MN patch. The patch is fixed between the upper and lower clamps, maintaining the grip. The upper jaw is dropped at a constant speed of 0.50 mm/s until the sample ruptures. The maximum force required for the rupturing and percentage elongation of the sample are estimated. The tensile strength is essential to determine the material’s extensibility [15].

#### 2.3.8. Insertion Study

The insertional studies were performed by inserting the MN array into Parafilm using a spring applicator. The Parafilm was folded to create an 8-layer stack of about 1 mm in overall thickness. The 10 × 10 array of MNs was inserted into the applicator to ensure the uniform force of application and inserted into the folded Parafilm stack. After the removal of MNs, the stack of Parafilm was examined under a microscope to obtain the number of holes formed. The percentage insertion efficiency is determined by the following formula [6].

$$Percent\;Insertion=\frac{Number\;of\;holes\;created\;in\;the\;Parafilm\;\times \;100}{Total\;No.\;of\;Microneedles}$$

#### 2.3.9. Analytical Method for Pregabalin Quantification

A UV–visible spectrophotometer (Shimadzu, Kyoto, Japan) was utilized to quantify the pregabalin. Based on the previously reported literature [18], the detection wavelength (λmax) was predetermined to be 225 nm. The standard pregabalin stock solution was produced using phosphate-buffered saline (PBS, pH 7.4) and was further diluted to obtain the working standard solution. The standard solutions were prepared in the range of 2–20 μg/mL to obtain the calibration curve. The absorbance of each solution was assessed at 225 nm against PBS set as a blank.

#### 2.3.10. Drug Loading Efficiency

Drug loading content is to examine the actual amount of drug present in the MN matrix after complete formulation. The MN patch was dissolved in 1 mL of phosphate-buffered saline (PBS, pH 7.4) using sonication for 30 min to ensure complete extraction of pregabalin from the polymer matrix. The sonicated solution is further filtered using a 0.2 µm membrane filter to remove any undissolved polymer residues. The filtrate is then examined using UV–visible spectroscopy to quantify the concentration of drug present in it. Drug content was determined using the validated calibration curve described in Section 2.3.9. The percentage drug loading efficiency was determined using the following formula. Theoretical drug content was calculated based on the initial amount of pregabalin added during formulation [19].

$$\%\;Drug\;Loading\;Efficiency\;=\;\frac{Actual\;drug\;content\;in\;microneedle\;patch\;\times \;100}{Theoretical\;drug\;content}$$

#### 2.3.11. Fourier Transform Infrared Spectroscopy (FTIR)

The SHIMADZU IRTracer-100 (range 400–4000 cm^−1^) was utilized to analyze the chemical structures of the drug and MN formulation, performing FTIR analysis. A small quantity of dried microneedle material was gently powdered using a mortar and pestle prior to FTIR analysis. Similarly, the pure drug and the polymer of the MN are also scanned at a resolution of 4 cm^−1^. The spectrum obtained of the pure drug and polymer is analyzed, and major peaks of the functional group are noted and compared to the spectrum resultant from the MN formulation. FTIR analysis is crucial to determine the compatibility of the drug incorporated in the MN formulation [5,6]. 

#### 2.3.12. Differential Scanning Calorimetry (DSC)

The DSC analysis was carried out to understand the nature of the drug and polymer in the MN matrix. It was conducted using differential scanning calorimetry (NETZSCH, Waldkraiburg, Germany). Nearly 5 mg of the sample is transferred to an aluminum pan and sealed. The samples are heated from about 25 °C to 150 °C at intervals of 10 °C per minute. Parallelly, to maintain an inert environment, dry nitrogen gas is flowed at 70 mL/min [20].

#### 2.3.13. X-Ray Powder Diffraction (XRD)

X-ray powder diffraction analysis is used to determine the phase changes and drug interactions in the powdered sample. The test was performed using an X-ray diffractometer (XRD) (powder and thin films) (PANalytical, Almelo, The Netherlands). The powdered sample was placed in a low-background sample holder and uniformly leveled to obtain the accurate diffraction. The sample is closed and run with a Cu Kα radiation (λ ≈ 1.5406 Å) X-ray source with a tube voltage of 40 kV and a current of 30–40 mA. The optimum scan range of 5° to 75° (2θ) was used to run a scan in a continuous mode of 0.02° 2θ per 0.5–1.0 s. The step size scan ensured better resolution, and time per step regulated the peak. Comparing the diffraction patterns of the pure drug, polymer, and MN formulation yielded an identification of the physical state of the sample and phase changes during the formulation [21,22].

#### 2.3.14. In Vitro Drug Release

The MN patches were dissolved in 50 mL PBS containing 3% *w*/*v* Tween 80 and maintained at 37 °C. Although pregabalin is water-soluble, Tween 80 (3% *w*/*v*) was added to maintain sink conditions and prevent drug adsorption, ensuring consistent drug release in the presence of polymeric matrices. Samples were collected at predetermined time intervals of 0.5, 1, 2, 4, 6, 8, 12, and 24 h. The total duration of the in vitro drug release study was 24 h, and the final sample was collected at 24 h.

The insertion and permeation behavior of the microneedle patches was evaluated using a Franz diffusion cell setup. Three layers of Parafilm^®^ were used as an artificial skin simulant to evaluate the insertion capability and penetration behavior of the microneedles. Multiple Parafilm layers are commonly employed to estimate insertion depth and mechanical penetration efficiency of microneedle arrays under controlled experimental conditions. The dissolution medium (PBS) maintained at 37 °C by circulating water through a peristaltic pump was poured into the receptor compartment and stirred at 600 rpm. The drug-loaded microneedle patches were placed onto the stacked Parafilm membrane between the donor and receptor compartments. At predetermined intervals, 0.5 mL samples were withdrawn from the sampling arm, and the amount of drug released was analyzed using UV–visible spectroscopy. The drug concentration (µg/mL) at each time point was calculated using the calibration curve and subsequently converted into cumulative percentage drug release [6,20]. Although Parafilm^®^ was utilized in the present study as a preliminary support membrane during the in vitro release setup, recent studies have indicated that Parafilm may not fully replicate the permeability characteristics of biological skin membranes. Therefore, further ex vivo permeation studies using animal or human skin models are required to comprehensively evaluate the transdermal delivery performance of the developed dissolving microneedle system.

All experiments were performed in triplicate (n = 3), and results are expressed as mean ± standard deviation.

## 3. Results and Discussion

### 3.1. Design and Fabrication of 3D-Printed Master Mold and Reverse PDMS Microneedle Molds

**Figure 4:**
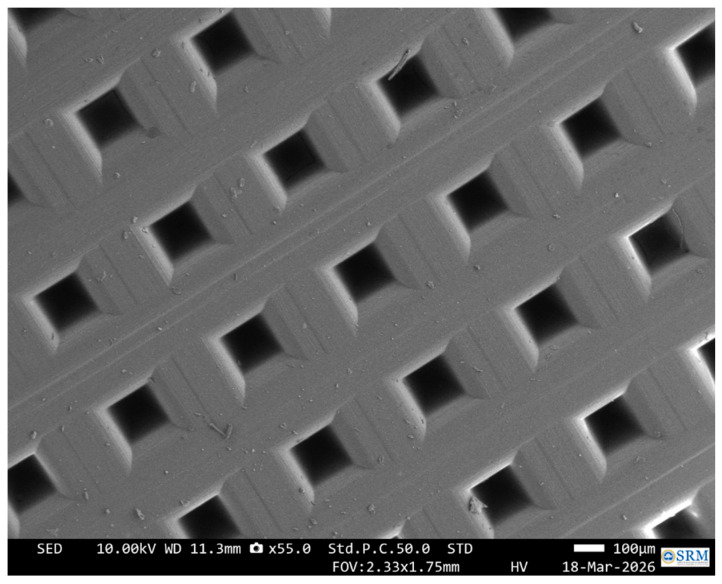
SEM image of PDMS microneedle Mold morphology.

The 3D-printed master micromold has been successfully printed and shows a well-defined structure with the same geometry as given. Following this, the negative microneedle mold was fabricated using polydimethylsiloxane (PDMS). The prepared PDMS negative mold exhibited the uniform cavity formation with sharp features and smooth surfaces, as shown in Figure 4. Therefore, the successful preparation of the 3D-printed master micromold and negative PDMS molds ensured reproducibility and consistency in the preparation of dissolving microneedle patches. The fabricated microneedles were further characterized using SEM analysis to evaluate their surface morphology and structural integrity.

### 3.2. Rheology Analysis (Viscosity)

**Figure 5:**
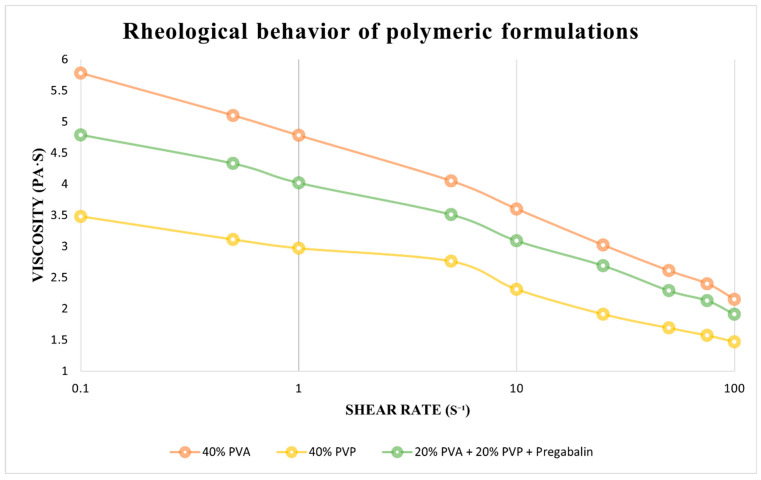
Rheological behavior of polymeric formulations showing viscosity as a function of shear rate (0.1–100 s^−1^). All formulations exhibited shear-thinning behavior, with viscosity decreasing as shear rate increased. Data are expressed as mean ± standard deviation (n = 3).

The rheological behavior of PVA, PVP, and pregabalin-loaded PVA/PVP polymeric solutions was evaluated over a shear rate range of 0.1–100 s^−1^. As illustrated in Figure 5, all formulations exhibited shear-thinning (pseudoplastic) behavior, characterized by a progressive decrease in viscosity with increasing shear rate. The viscosity of the PVA solution decreased from 5.78 Pa·s at 0.1 s^−1^ to 2.15 Pa·s at 100 s^−1^, indicating strong intermolecular hydrogen bonding and polymer chain entanglement. In contrast, the PVP solution showed lower viscosity values, decreasing from 3.48 Pa·s to 1.47 Pa·s, which can be attributed to its relatively flexible polymeric structure. The pregabalin-loaded PVA/PVP formulation exhibited intermediate viscosity values, ranging from 4.79 Pa·s to 1.91 Pa·s, indicating a balanced rheological profile. This behavior facilitates efficient mold filling at higher shear rates while maintaining sufficient viscosity at lower shear rates to preserve microneedle structural integrity. Rheological analysis was important to evaluate the flow behavior and viscosity characteristics of the polymeric solutions, as these parameters directly influence mold filling efficiency, microneedle formation, and fabrication reproducibility during the casting process. Polymer concentrations of 40% were selected for rheological evaluation because lower concentrations such as 20% exhibited comparatively lower viscosity and insufficient mechanical strength, which may adversely affect proper mold filling and structural integrity of the fabricated microneedles. The rheological profile is consistent with the graphical representation in Figure 5.

### 3.3. Morphology (SEM)

**Figure 6:**
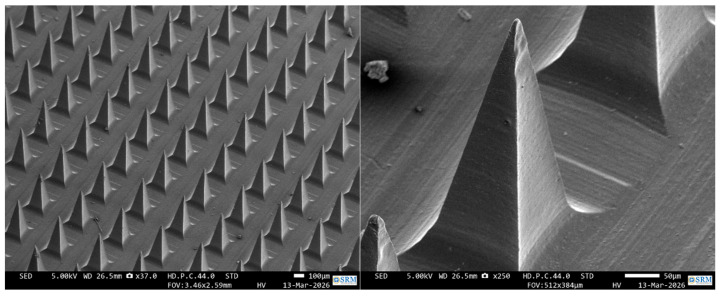
SEM images of dissolving microneedle arrays.

The well-structured morphology of designed pregabalin-loaded PVA/PVP dissolving microneedles was examined using SEM analysis. They were observed to exhibit a pyramidal structure with sharp tips resembling the designed dimensions, as shown in Figure 6. The geometric characteristics of the fabricated microneedles were analyzed from SEM images and were found to be consistent with the designed parameters. The microneedles exhibited an approximate height of 850 µm, a base radius of 200 µm, and a tip radius of approximately 15 µm. The microneedle array consisted of a 10 × 10 configuration with an interspacing of approximately 450 µm between adjacent needles, indicating uniform distribution across the patch. These results confirm the accuracy of the 3D-printing-assisted mold fabrication process and the structural uniformity of the microneedle arrays. The fabrication potency was also denoted by the absence of cracks and deformations in the structure, which ensured robust mold replication and uniform polymer distribution. These properties are necessary to retain competent skin transportation, physical stability, and a consistency in the drug delivery process; moreover, they regulate the developed microneedle for transdermal application.

Scanning electron microscopy (SEM) images illustrating the morphology of fabricated microneedles showed uniform pyramidal structures with sharp tips, smooth surfaces, and absence of structural defects such as cracks or deformation.

### 3.4. Uniformity of Thickness, Width and Weight Variation

The formulated dissolving microneedles show excellent geometrical dimensions and weight uniformity among all three patches evaluated. As shown in Table 1, the measured values closely correspond to the specifications of designed microneedle patches. The minimal variability noticed among all three patches shows that the micromolding method using 3D-printed molds provided even polymer distribution and uniform microneedle formulation. Such uniformity is essential for consistent drug loading, mechanical stability, and consistent drug delivery performance, thereby enhancing the overall performance of microneedle drug delivery systems.

### 3.5. Folding Endurance

The folding endurance test was performed to assess the flexibility and mechanical stability of the microneedle patch. The formulated dissolving microneedle indicates excellent mechanical flexibility properties and resistance to rupture. As shown in Table 1, the average folding endurance value is 214 folds. The significant number of folds needed to break the polymeric matrix provides excellent elasticity and mechanical strength. This flexibility provides the microneedle patches with the ability to resist handling and application without structural damage. As a result, the fabricated patches show adequate mechanical strength for effective microneedle drug delivery applications.

### 3.6. Swelling Study

The swelling study was conducted to evaluate the hydration behavior of the polymer matrix, which influences drug release and microneedle dissolution. The drug-loaded dissolving microneedle shows significant swelling behavior in PBS 5.5, which replicates the pH of the skin. As presented in Table 1, the swelling index mean value is 44.5 ± 0.7%. The resulting swelling index indicates that both polymers (PVA and PVP) are hydrophilic in nature and therefore easily absorb moisture and undergo hydration. This swelling index improves polymer dissolution and drug release after administration into the skin; therefore, it improves the efficacy of fabricated dissolving microneedle patches.

### 3.7. Mechanical Strength (Axial Compression)

The pregabalin-loaded dissolving microneedle arrays displayed excellent mechanical strength under axial compression, with an average force value of 3.3 ± 0.15 N/needle, as shown in Table 1. The percentage height reduction of the microneedles after compression was found to be approximately 5–10%, indicating minimal deformation under applied force and confirming good mechanical integrity of the microneedle structure. The resulting compression force is significantly higher than the required minimal force for skin penetration (0.1 N/needle), which shows that the fabricated dissolving microneedles have adequate mechanical strength for penetration of the stratum corneum without mechanical failure, thus showing their suitability for effective microneedle drug delivery applications [23].

### 3.8. Tensile Strength

The formulated dissolving microneedle shows adequate tensile strength, with a mean value of 3.21 ± 0.03 MPa, as shown in Table 1. The results show that the mechanical strength of the patches is due to the film-forming potential and intermolecular interactions between the polymers used, which improves the elasticity and structural strength of the backing layer. Therefore, mechanical strength is essential to withstand mechanical stress during handling, storage, and application, thereby ensuring the structural integrity of the dissolving microneedle array for effective drug delivery.

### 3.9. Insertion Study

**Figure 7:**
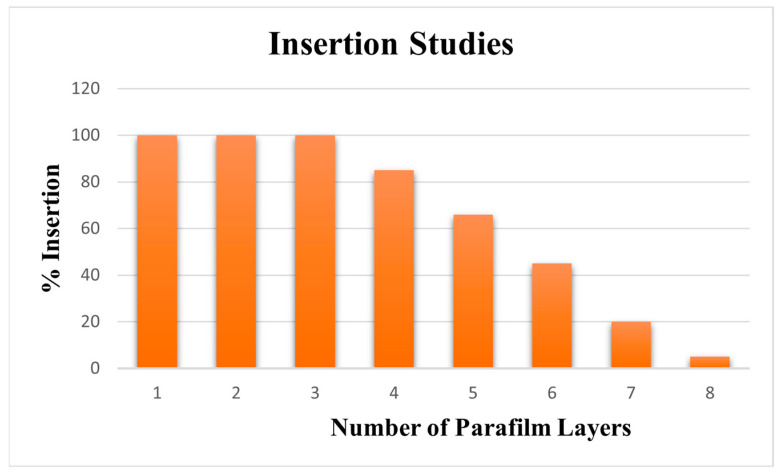
Insertion efficiency of microneedles using Parafilm model.

The insertion percentage of the prepared dissolving microneedles was examined using a Parafilm model (eight layers) to mimic skin penetration. Parafilm was selected as a model membrane due to its multilayered structure, which provides mechanical resistance similar to that of the stratum corneum. Each Parafilm layer (~130 µm thickness) acts as a barrier, allowing indirect assessment of microneedle insertion depth by counting the number of penetrated layers. This model has been widely used as a cost-effective and reproducible alternative to biological skin for evaluating microneedle penetration efficiency. The layered arrangement of Parafilm mimics the barrier function of the stratum corneum by providing resistance to needle insertion, thereby enabling estimation of penetration capability [24]. The geometrical structure of 100 pyramidal microneedles (10 × 10 arrays) with a height of 850 µm was inserted into the eight-layer Parafilm for 30 s. The thickness of each Parafilm layer is about 130 µm, which gives a cumulative thickness of 1040 µm for all eight layers. As shown in Figure 7, the results of insertion studies show that all the microneedles penetrated 100% till the 3rd layer of Parafilm, at a depth around 390 µm, whereas about 85% of needles penetrated the 4th layer, showing deeper penetration into the skin model membrane. These results indicate that the prepared microneedles exhibit sufficient mechanical strength and an optimal structure to penetrate the barrier layer and produce microchannels; therefore, they are suitable for effective microneedle drug delivery applications.

A bar graph representing the percentage insertion of microneedles across multiple layers of Parafilm, indicating effective penetration into deeper layers and demonstrating adequate mechanical strength, was made. Data are expressed as mean ± standard deviation (n = 3).

### 3.10. Analytical Method for Pregabalin Quantification

**Figure 8:**
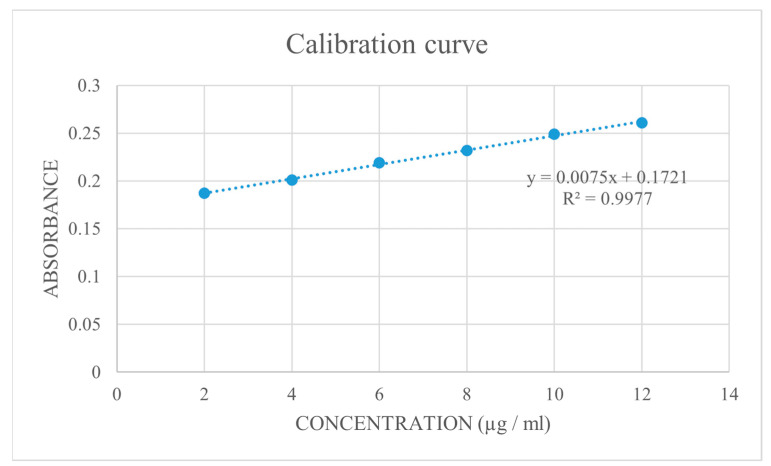
Calibration curve of pregabalin showing linear relationship between concentration (2–12 µg/mL) and absorbance at 225 nm.

The proportional correlation between concentration and absorbance was achieved by obtaining an efficient linearity calibration curve of pregabalin over the range of 2–12 μg/mL. As shown in Figure 8, the regression equation was expressed as y = 0.0075x + 0.1721 along with a correlation coefficient of R^2^ = 0.9977, denoting a strong linear correlation. The calibration curve demonstrated a strong linear relationship between concentration and absorbance, indicating its suitability for quantitative estimation of pregabalin in the microneedle formulation.

### 3.11. Drug Loading Efficiency

The drug loading efficiency is studied to determine the potency of pregabalin incorporated in the polymer matrix of dissolving microneedles. The analysis displayed a drug loading efficiency of 92.4 ± 0.20%, which indicated robust entrapment of pregabalin leading to good solubility. The high drug loading efficiency also indicates uniform distribution of pregabalin within the polymeric casting solution, thereby contributing to homogenous distribution of the drug into the microneedle matrix. These findings suggest efficient incorporation of pregabalin within the PVA/PVP polymeric matrix with minimal drug loss during fabrication, thereby supporting the suitability of the formulation for dissolving-microneedle-based drug delivery. All values are expressed as mean ± standard deviation (n = 3).

### 3.12. Fourier Transform Infrared Spectroscopy (FTIR)

**Figure 9:**
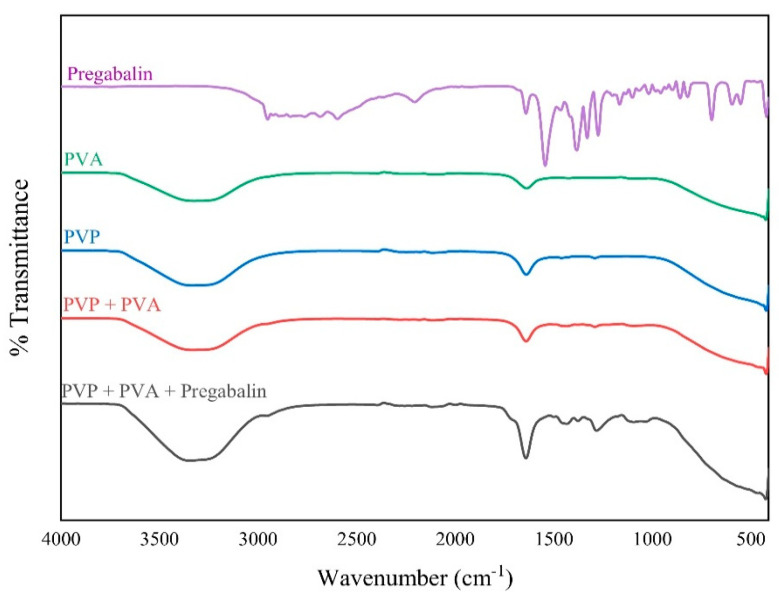
FTIR spectra of drug, polymers, and microneedle formulation.

FTIR spectra were recorded for the pure drug (pregabalin), PVA, PVP, dissolving microneedles without drug (PVA/PVP), and drug-loaded dissolving microneedles (PVA/PVP + pregabalin). The spectral range of 4000–400 cm^−1^ was used to record % transmittance. The FTIR spectrum of pure pregabalin showed characteristic peaks at ~3300–3400 cm^−1^, attributed to N–H stretching vibrations of the primary amine group along with contributions from aliphatic C–H stretching; ~1650–1600 cm^−1^, corresponding to C=O stretching of the carboxylic acid group; ~1450–1400 cm^−1^, assigned to CH_2_ bending vibrations; and ~1100–1000 cm^−1^, corresponding to C–N stretching vibrations, as shown in Figure 9. The observed functional group peaks are consistent with previously reported spectroscopic and solid-state characteristics of pregabalin [25]. The PVA spectrum exhibited a broad and intense absorption band around 3325–3400 cm^−1^ corresponding to O–H stretching vibrations, along with peaks around ~2940 cm^−1^ (C–H stretching) and ~1140–1090 cm^−1^ (C–O stretching). The PVP spectrum showed a prominent peak near ~1639–1650 cm^−1^ corresponding to C=O stretching of the lactam group, along with C–H stretching around ~2950 cm^−1^. The FTIR spectra of PVA and PVP exhibited partial overlap, particularly in the region around 1600–1650 cm^−1^, due to the presence of similar functional groups such as hydroxyl and carbonyl groups. This overlap may give the appearance of similar spectral profiles in the superimposed spectra; however, characteristic peaks of each polymer were identifiable. The dissolving microneedle without drug and the drug-loaded dissolving microneedle showed retention of the characteristic peaks of both the polymer and drug with slight shifts and changes in intensity, indicating the absence of chemical incompatibility between the polymers and pregabalin during fabrication. The observed minor variations in peak position and intensity may be attributed to intermolecular hydrogen bonding interactions between the carbonyl groups of PVP, hydroxyl groups of PVA, and functional groups of pregabalin. These spectral results confirm the compatibility and stability of pregabalin within the PVA–PVP polymer system and support its successful incorporation and molecular dispersion within the dissolving microneedle matrix.

Fourier transform infrared (FTIR) spectra of pure pregabalin, PVA, PVP, blank microneedles, and drug-loaded microneedles confirm chemical compatibility and absence of significant drug–polymer interactions.

### 3.13. Differential Scanning Calorimetry (DSC)

**Figure 10:**
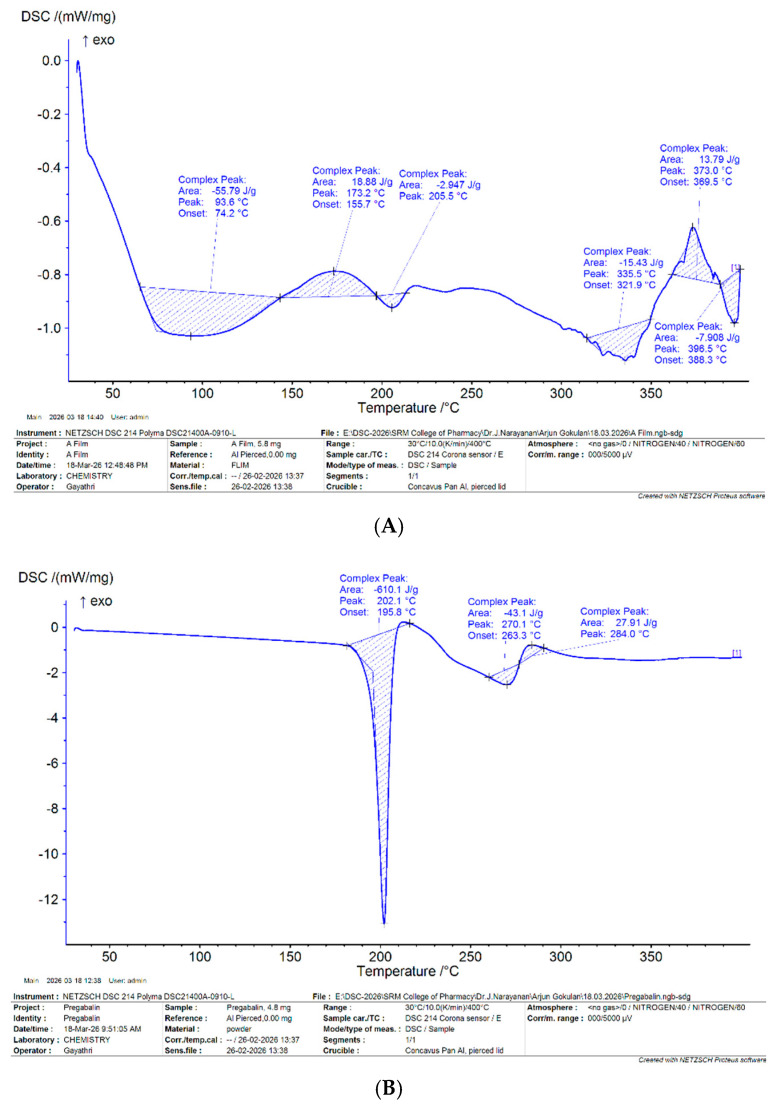
DSC thermograms of (**A**) pure pregabalin and (**B**) pregabalin-loaded PVA/PVP microneedle formulation. The characteristic melting endotherm of pregabalin observed in the pure drug thermogram showed reduced intensity and broadening in the microneedle formulation, indicating partial amorphization and molecular dispersion of pregabalin within the polymer matrix.

Differential scanning calorimetry (DSC) analysis was performed for pure pregabalin and pregabalin-loaded dissolving microneedles to evaluate the thermal behavior and physical state of the drug within the polymeric matrix. The DSC thermogram of pure pregabalin exhibited a sharp endothermic melting peak at 202.1 °C (onset: 195.8 °C), confirming its crystalline nature, as shown in Figure 10A. The DSC thermogram of the pregabalin-loaded dissolving microneedles, shown in Figure 10B, exhibited a broad endothermic transition around 93.6 °C, which may be attributed to moisture evaporation and relaxation behavior of the PVA/PVP polymeric matrix. Additional broadened thermal transitions were observed around 173.2 °C and 205.5 °C with reduced intensity compared to the pure drug thermogram. The reduction in sharpness and intensity of the characteristic pregabalin melting peak suggests partial reduction in crystallinity and possible molecular-level dispersion of the drug within the polymer matrix. No additional unexpected thermal peaks were observed, indicating the absence of significant drug–polymer incompatibility during formulation development. These findings support the successful incorporation of pregabalin into the PVA/PVP dissolving microneedle system.

### 3.14. X-Ray Powder Diffraction (XRD)

**Figure 11:**
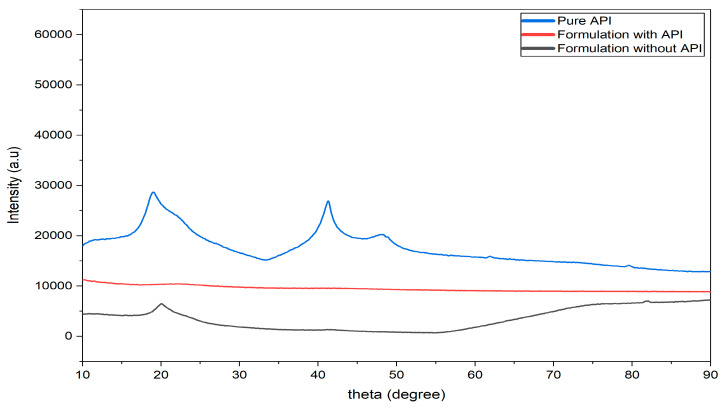
X-ray diffraction (XRD) patterns.

X-ray diffraction (XRD) analysis was performed to evaluate the crystalline nature of pure pregabalin and its physical state after incorporation into the PVA/PVP polymeric formulation. The diffraction pattern of pure pregabalin exhibited characteristic sharp diffraction peaks, particularly around 19–22° and 41–42° (2θ), confirming its crystalline nature, as shown in Figure 11. These findings are consistent with previously reported crystalline characteristics of pregabalin in the literature [25,26,27].

The pregabalin-free dissolving microneedle formulation exhibited a broad diffuse halo pattern without distinct crystalline peaks, indicating the amorphous nature of the PVA/PVP polymer matrix. In contrast, the pregabalin-loaded dissolving microneedle formulation showed a marked reduction in the intensity of characteristic pregabalin diffraction peaks along with peak broadening when compared with pure pregabalin. The reduction in peak intensity and broadening of peaks suggest partial amorphization and molecular dispersion of pregabalin within the polymeric matrix. Furthermore, no additional diffraction peaks were observed, indicating the absence of significant drug–polymer incompatibility during formulation development. These results confirm the successful incorporation of pregabalin into the PVA/PVP dissolving microneedle system and support improved drug dispersion within the polymer matrix.

Superimposed XRD patterns of pure pregabalin (API), PVA/PVP formulation without API, and pregabalin-loaded dissolving microneedle formulation were made. Characteristic diffraction peaks of pregabalin show reduced intensity and peak broadening in the formulation, indicating partial amorphization and successful incorporation of the drug within the polymer matrix.

### 3.15. In Vitro Drug Release

**Figure 12:**
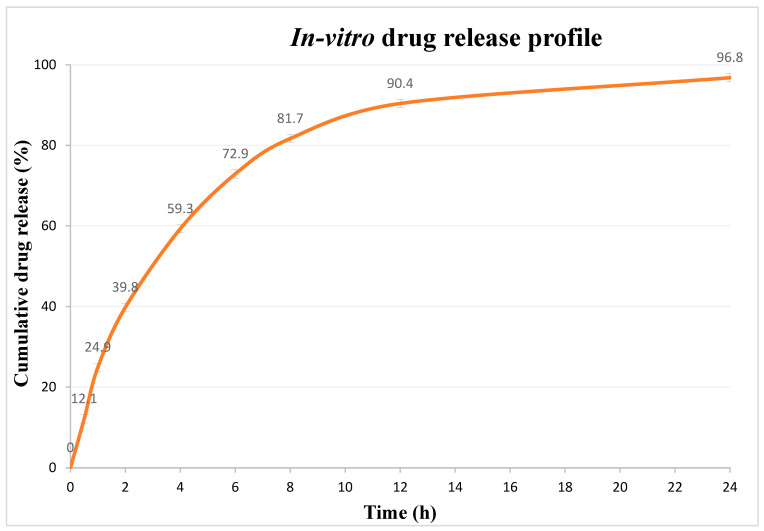
In vitro drug release profile.

The in vitro drug release profile of pregabalin-loaded PVA/PVP dissolving microneedles featured sustained release of drug for a period of 24 h following rapid release in the initial hours. As shown in Figure 12, the results show the formulation releasing 12.1% of pregabalin in 0.5 h, subsequently increasing to 24.90% at 1 h, 39.80% in 2 h, and 59.30% in 3 h, reflecting the primary drug elution from the microneedle matrix. The aggregated release was further observed at 6 h, increasing to 72.90%; at 8 h, to 81.70%; and at 12 h, to 90.40%, escalating to 96.80% at 24 h. This pattern of release showcased rapid hydration and dissolution of the hydrophilic PVA/PVP matrix, ensuring efficient pregabalin release over 24 h and highlighting its potential applicability for dissolving-microneedle-based drug delivery. Pure pregabalin, due to its high aqueous solubility, typically exhibits rapid dissolution with near-complete drug release within a short duration. In contrast, the fabricated dissolving microneedle shows a biphasic release profile characterized by an initial burst release followed by sustained drug release over 24 h. Recent studies have also reported pregabalin-loaded dissolving microneedle systems for neuropathic pain management [3]. They demonstrated the successful development of pregabalin-loaded dissolving microneedles with effective drug delivery characteristics for peripheral neuropathic pain applications. The findings of the present study are consistent with these previously reported outcomes, particularly regarding efficient drug incorporation, structural integrity, and sustained drug release behavior. However, the current study further emphasizes the use of a 3D-printing-assisted mold fabrication approach combined with a bilayer PVA/PVP polymeric system to improve fabrication reproducibility, geometrical precision, and controlled biphasic drug release characteristics. This sustained release behavior can be attributed to the hydrophilic polymeric matrix (PVA/PVP), which regulates drug diffusion and prolongs drug release from the microneedle system. All values are expressed as mean ± standard deviation (n = 3). The half-life (t50%) of drug release was approximately 3.0 h, indicating a controlled and sustained release behavior of the formulation.

Cumulative percentage drug release of pregabalin from dissolving microneedles over 24 h, demonstrating an initial burst release followed by sustained drug release behavior. Data are expressed as mean ± standard deviation (n = 3).

## 4. Conclusions

In this current research study, pregabalin-loaded PVA-PVP dissolving microneedle patches were successfully prepared using a 3D-printed-based micromolding technique. The SEM analysis shows the fabricated microneedles exhibit pyramidal structures with high-precision, uniform dimensions and excellent morphological structures. The formulation exhibited the good viscosity properties for filling the mold, along with ideal mechanical strength, uniformity, swelling index, and flexibility. This confirms the structural stability during handling and application. The mechanical test indicates that the microneedles have excellent axial compression strength, which confirms that microneedles penetrate the stratum corneum easily.

Drug loading efficiency evaluation shows that pregabalin is well incorporated into dissolving microneedles with a drug loading efficiency of 92.4%, which signifies the minimal loss of drug. Differential scanning calorimetry and Fourier transform infrared spectroscopy examination confirm the chemical/thermal stability and compatibility of pregabalin within the PVA-PVP polymer matrix systems, with no signs of chemical interactions. X-ray powder diffraction shows a significant decrease in crystalline nature and partial amorphization of pregabalin, which confirms the pregabalin was successfully distributed in the PVA-PVP polymer matrix, and in vitro drug release shows a sustained drug release profile.

The developed system exhibited desirable mechanical, physicochemical, and drug release properties, highlighting its potential applicability as a dissolving-microneedle-based drug delivery platform. However, further ex vivo and in vivo permeation studies using biological skin models are required to comprehensively validate the transdermal delivery performance of the developed dissolving microneedle system. This research study shows the crucial role of 3D-printing-assisted mold development to improve the reliability, reproducibility, and scalability of microneedle-based drug delivery systems.

## Figures and Tables

**Table 1:** Physicochemical and mechanical characterization of pregabalin-loaded dissolving microneedles (n = 3).

Parameter	Result
Thickness (mm)	0.50 ± 0.005
Width (mm)	10.00 ± 0.01
Weight (mg)	18.1 ± 0.1
Folding Endurance (Number of folds)	214 ± 4
Swelling Index (%)	44.5 ± 0.7
Compression Force (N/needle)	3.3 ± 0.15
Tensile Strength (MPa)	3.21 ±0.03

## Data Availability

The original contributions presented in this study are included in the article. Further inquiries can be directed to the corresponding author.

## Abbreviations

MN	Microneedle
DMN	Dissolving Microneedle
PDMS	Polydimethylsiloxane
PVA	Polyvinyl Alcohol
PVP	Polyvinylpyrrolidone
API	Active Pharmaceutical Ingredient
CAD	Computer-Aided Design
STL	Stereolithography File Format
SLA	Stereolithography Apparatus
SEM FESEM	Scanning Electron Microscopy/Field Emission Scanning Electron Microscopy
FTIR	Fourier Transform Infrared Spectroscopy
DSC	Differential Scanning Calorimetry
XRD	X-ray Diffraction
PBS	Phosphate-Buffered Saline
CMC	Carboxymethyl Cellulose
UV–Vis	Ultraviolet–Visible
ATR	Attenuated Total Reflectance
rpm	Revolutions Per Minute
°C	Degree Celsius
µm	Micrometer
mm	Millimeter
MPa	Megapascal
N	Newton
Pa·s	Pascal-second (viscosity unit)
*w*/*w*	Weight by Weight
pH	Potential of Hydrogen
